# 1417. Continued Success of *Legionella* Water Screening for a Large Veterans Affairs Campus in Long Island, New York

**DOI:** 10.1093/ofid/ofad500.1254

**Published:** 2023-11-27

**Authors:** Lisa Bailey, Mahfuz Rahman, Debra Belanger, Monique Thorne, Florence M Ford, Mark Gouch, George Psevdos

**Affiliations:** Northport VAMC, Northport, New York; Renaissance School of Medicine at Stony Brook University, Corona, New York; Northport VAMC, Northport, New York; Northport VAMC, Northport, New York; Northport VA, Northport, New York; Northport VAMC, Northport, New York; Northport VA Medical Center, Northport, New York

## Abstract

**Background:**

*Legionella* is found in all natural water sources & has been linked to waterborne environmental outbreaks of pneumonia. Environmental surveillance in healthcare facilities is of paramount importance to prevent *Legionella* overgrowth and acquired infections. This can a be a challenging task for our large 268-acre Veterans Affairs campus with its own water supply wells and water distribution system, 10 buildings housing acute care, outpatient clinics, residential and nursing homes. While the COVID 19 pandemic absorbed resources & time, water testing was not neglected. We report our experience of *Legionella* water testing during the pandemic

**Methods:**

Water *Legionella* sample testing is done quarterly involving 180 faucets & showers throughout the facility, ice machines and 4 cooling towers (CT). Cultures are performed in a reference laboratory. Annual cost $100,000. A positive detection is defined as 1 colony forming unit (CFU)/mL from faucets and 10 CFU/mL from CT. Every positive detection is managed as follows: remediation by hyperchlorination, resampling, disinfection of cooling towers, and removal of equipment (ice machines)

**Results:**

In fiscal Years 2020-22 4853 Veterans were hospitalized and 455 were tested for *Legionella* via urinary antigen; 3/455 patients had legionellosis, all were community-related, & 1 had a concurrent coinfection with COVID-19. 2221 water samples were collected. Table 1 shows detections and locations. All detections were remediated by localized chlorine disinfection & then resampled. Retreatments with increased disinfectant dosage was implemented in repeat positive detections. In few instances up to 3 retreatments were required to achieve negative growth. For repeat positive detections for CTs remediation utilized addition of a 2nd biocide No employee/patient case of *Legionella* pneumonia has been associated with exposure to positive water testing

**Table 1**

**Figure:**
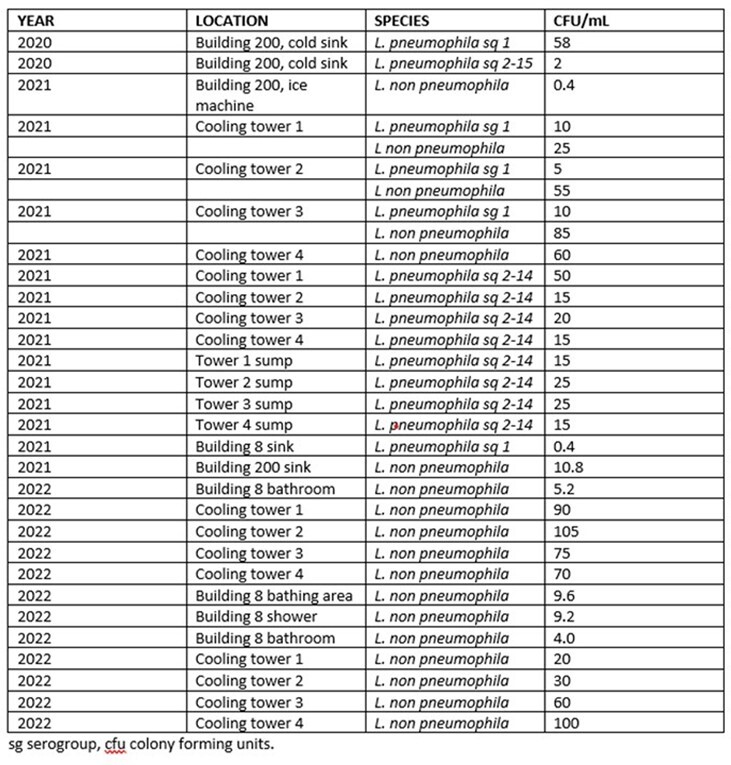

Positive Legionella detections

**Figure 1**

**Figure:**
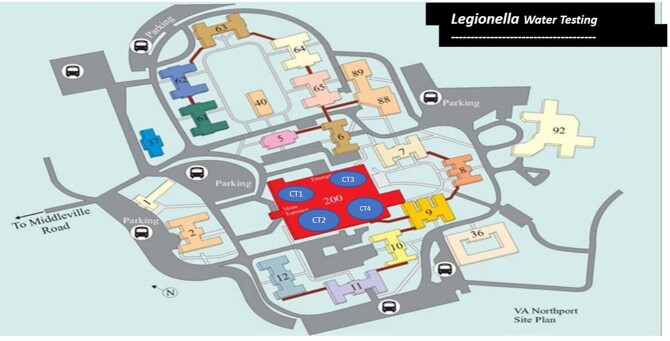

Facility Diagram location of buildings and Cooling Towers

**Conclusion:**

Preventing *Legionella* water overgrowth in a large Veteran Affairs campus can be a tedious and costly endeavor. *L. pneumophila and non pneumophila* species were detected in various locations including the CTs, nevertheless remediation protocols were immediately followed and proved successful. There were only 3 cases of *Legionella* pneumonia in the study period, but none were facility associated

**Disclosures:**

**All Authors**: No reported disclosures

